# Oxidative stress and adrenocortical insufficiency

**DOI:** 10.1530/JOE-13-0346

**Published:** 2014-06

**Authors:** R Prasad, J C Kowalczyk, E Meimaridou, H L Storr, L A Metherell

**Affiliations:** 1 Barts and the London School of Medicine and Dentistry, William Harvey Research Institute, Centre for Endocrinology, Queen Mary University of London John Vane Science Centre, Charterhouse Square, London, EC1M 6BQ UK

**Keywords:** oxidative stress, reactive oxygen species, steroidogenesis, adrenal insufficiency

## Abstract

Maintenance of redox balance is essential for normal cellular functions. Any perturbation in this balance due to increased reactive oxygen species (ROS) leads to oxidative stress and may lead to cell dysfunction/damage/death. Mitochondria are responsible for the majority of cellular ROS production secondary to electron leakage as a consequence of respiration. Furthermore, electron leakage by the cytochrome P450 enzymes may render steroidogenic tissues acutely vulnerable to redox imbalance. The adrenal cortex, in particular, is well supplied with both enzymatic (glutathione peroxidases and peroxiredoxins) and non-enzymatic (vitamins A, C and E) antioxidants to cope with this increased production of ROS due to steroidogenesis. Nonetheless oxidative stress is implicated in several potentially lethal adrenal disorders including X-linked adrenoleukodystrophy, triple A syndrome and most recently familial glucocorticoid deficiency. The finding of mutations in antioxidant defence genes in the latter two conditions highlights how disturbances in redox homeostasis may have an effect on adrenal steroidogenesis.

## Introduction

Reactive oxygen species (ROS) are derived from O_2_ and comprise molecules with varying oxidant properties. At low concentrations, ROS modulate many cellular processes through redox-dependent signalling, including proliferation, differentiation, apoptosis, immune regulation and cellular adaptation to stress (Ray *et al*. 2012, Sena & Chandel 2012). A critical balance in redox status needs to be maintained and this is achieved by numerous interacting antioxidant pathways. Oxidative stress occurs when this balance is disturbed. ROS can then have deleterious effects on proteins, lipids and nucleic acids, ultimately leading to cell damage and death. Oxidative stress is implicated in a plethora of conditions including neurodegenerative disorders, diabetes mellitus, cardiovascular disease and ageing. Antioxidant defence mechanisms are complex and can be specific to cell type and furthermore to sub-cellular compartment. In comparison to many other tissues, including those with high metabolic demand such as the liver and brain, the adrenal cortex has high levels of several antioxidants, both enzymatic and non-enzymatic. This investment is necessary given the high turnover of lipid within the mitochondria and ROS production during steroidogenesis. Disturbances in redox homeostasis within the adrenocortical environment may therefore have an effect on steroidogenesis, as evidenced by several disorders of adrenal insufficiency, including most recently familial glucocorticoid deficiency (FGD).

## ROS generation in mitochondria

**Figure 1:**
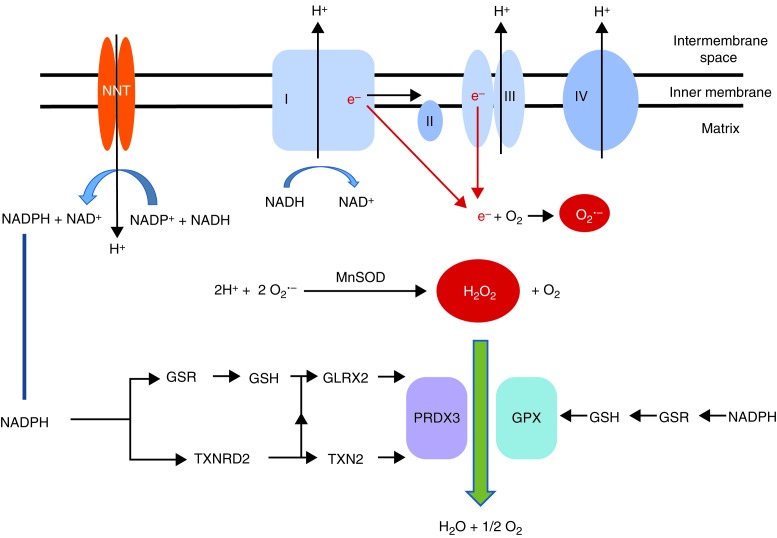
Detoxification of mitochondrial superoxide species produced during electron leakage from the mitochondrial electron transport chain. The superoxide radical O_2_ · ^−^, produced from electrons (e^−^) leaked at complexes I and III of the mitochondrial electron transport chain, can be protonated to form H_2_O_2_, this process of dismutation is catalysed by MnSOD. Mitochondrial H_2_O_2_ is detoxified by the thioredoxin and GSH systems, which require high concentrations of nicotinamide adenine dinucleotide phosphate (reduced NADPH) provided by NNT. The flow of electron transfer from NADPH to PRDX3 and GPX, through the various components of the thioredoxin and GSH systems, is shown in the figure. GSR, glutathione reductase; GSH, reduced glutathione; TXNRD2, thioredoxin reductase 2; TXN2, thioredoxin 2; GLRX2, glutaredoxin 2; NNT, nicotinamide nucleotide transhydrogenase; PRDX3, peroxiredoxin 3; GPX, glutathione peroxidase; MnSOD, manganese superoxide dismutase.

Mitochondria are responsible for the majority of cellular ROS production secondary to electron leakage as a consequence of respiration. During respiration, electrons are transferred across to molecular oxygen to generate water via the four complexes of the electron transport chain. The electron–proton gradient established during this process is used to generate energy in the form of ATP. As a result of electron leakage at complex I (NADH dehydrogenase) and complex III (cytochrome *c* reductase), a small percentage of the molecular oxygen is converted to superoxide anions (O_2_ · ^−^) (Fig. 1). These superoxide anions can be protonated to form strongly oxidant hydroxyl radicals (·OH) which can cause oxidative modification of proteins and membrane lipids. ROS can also modify key components of the electron transport chain thereby further exacerbating electron leakage and superoxide production. Dismutation, catalysed by the superoxide dismutases (SODs), reduces superoxide ions to hydrogen peroxide (H_2_O_2_). H_2_O_2_ can also contribute to oxidative stress by reacting with free metals to form OH. In addition, in comparison to O_2_ · ^−^, which tends to remain at the site of production, H_2_O_2_ readily traverses membranes into other subcellular compartments where it may affect signalling pathways.

Several compartment-specific antioxidant mechanisms are in place to target superoxide production. Cytoplasmic copper/zinc-dependent SOD1 (CuZnSOD), mutations of which are associated with the neurodegenerative disorder, amyotrophic lateral sclerosis, and mitochondrial matrix manganese-dependent SOD2 (MnSOD) catalyse the conversion of superoxide anions into H_2_O_2_ (Fig. 1). H_2_O_2_ has preferential reactivity towards (seleno) cysteine (Cys) residues in target proteins, and is detoxified by members of the glutathione peroxidase (GPX) and peroxiredoxin (PRDX) families, discussed later in this review. The relative contribution of each of these enzymes may be tissue- and compartment-specific and determined by the source of H_2_O_2_ and the expression levels of each individual enzyme.

**Figure 2:**
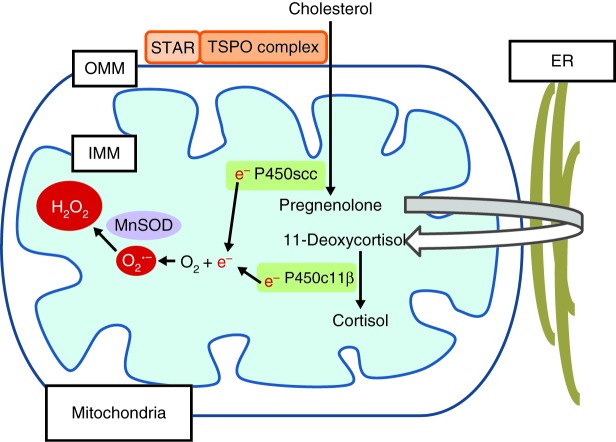
Reactive oxygen species (ROS) production during steroidogenesis. The import of cholesterol from the outer mitochondrial membrane (OMM) to the inner mitochondrial membrane (IMM) is activated by STAR and mediated by the translocator protein (TSPO)-associated multi-component complex. This is followed by cholesterol side-chain cleavage to pregnenolone by P450scc (CYP11A1). The other steps in the steroidogenic pathway are catalysed by cytochrome P450 isoforms in the endoplasmic reticulum (ER). The final step in cortisol synthesis is catalysed by P450c11β (CYP11B1) in the mitochondria, converting 11-deoxycortisol to cortisol. Electron leakage during this process results in ROS production.

In addition to electron leakage during respiration, other sources of superoxide production within the mitochondria include reactions catalysed by xanthine/xanthine oxidase, uncoupled nitric oxide synthases, NADPH-dependent oxidases (NOXs) and, most pertinent to the adrenal cortex, cytochrome P450 isoforms. The P450 enzymes are involved in the biosynthesis of cholesterol-derived steroidal compounds (Miller 2005). In steroidogenic tissues, the levels of expression of P450 enzymes are approximately ten times higher than those of the other electron transport chain proteins and electron leakage in the P450 system can also contribute to ROS production (Hanukoglu & Hanukoglu 1986, Hanukoglu 2006). P450 groups are represented by microsomal cytochrome P450s present in the endoplasmic reticulum, and mitochondrial cytochrome P450s present in the inner mitochondrial membrane. Type 1 P450 isoforms present in the mitochondria include P450scc (side-chain cleavage; CYP11A1), which catalyses the conversion of cholesterol to pregnenolone, and P450c11β (CYP11B1) responsible for the β-hydroxylation of 11-deoxycortisol to cortisol (Fig. 2). The reactions catalysed by these P450 enzymes require NADPH as a reducing agent, in addition to molecular oxygen. NADPH donates two electrons which are transferred to P450 enzymes via two electron transfer proteins, ferredoxin reductase (adrenodoxin reductase) and then ferredoxin (adrenodoxin) (Ziegler *et al*. 1999, Grinberg *et al*. 2000). Ferredoxin reductase is a flavin adenine dinucleotide, containing flavoenzyme and is able to accept both electrons from NADPH. These are then passed one at a time to ferredoxin and finally to the P450 enzymes for hydroxylation of substrates, the order of electron transfer is as follows (Hanukoglu 2006):

NADPH→ferrodoxin reductase→ferrodoxin→P450

In a tightly coupled system, all electrons from NADPH are used in substrate hydroxylation; however, electron transfer within the P450 system can be relatively ‘uncoupled’ or ‘leaky’, with the rate of electron leakage varying depending on P450 subtype (Hanukoglu 2006). Up to 40% of total electron flow in the P450c11β system, catalysing the final step in cortisol synthesis, is directed to ROS formation in comparison to 15% in the P450scc system (Rapoport *et al*. 1995). Thus steroidogenesis, particularly glucocorticoid production, contributes significantly to cellular ROS production (Fig. 2). Type 2 microsomal P450 isoforms, catalysing all the other steps in steroidogenesis, are present in the endoplasmic reticulum and are reliant on P450 oxidoreductase and NADPH for reduction (Miller 2005).

## Adrenal cortex antioxidant defence mechanisms

The adrenal cortex is particularly equipped to handle the increased risk of oxidative stress. Several antioxidants, both enzymatic and non-enzymatic, are highly expressed in comparison to other tissues. Ascorbic acid (vitamin C), which recycles α-tocopherol (vitamin A) radicals, is present at the highest levels in the adrenal cortex (Hornig 1975), indeed endogenous ascorbic acid was first isolated from adrenal tissue (Svirbely & Szent-Gyorgyi 1932). Depletion of ascorbic acid secondary to vitamin A deficiency in rats leads to adrenocortical degeneration (Gruber *et al*. 1976). Other non-enzymatic antioxidants present at high levels within the adrenal cortex include vitamins A and E (Azhar *et al*. 1995).

As previously described, O_2_ · ^−^ radicals are converted by SODs to H_2_O_2_. Mitochondrial H_2_O_2_ is further reduced by two major thiol antioxidant systems dependent on reduced glutathione (GSH) and the small protein thioredoxin 2 (TXN2), each system has several associated proteins (Fig. 1). Reduced GSH and components of the TXN2 system are seen in abundance in the adrenal cortex (Azhar *et al*. 1995, Watabe *et al*. 1999). Both TXN2 and GSH systems are dependent on the reducing power of NADPH. NADPH supply within the mitochondria is maintained by the proton pump nicotinamide nucleotide transhydrogenase (NNT). Mutations in *NNT* have recently been associated with FGD, discussed later in this review (Meimaridou *et al*. 2012). Mitochondrial NADPH stores can also be restored by mitochondrial dehydrogenases including malate dehydrogenase and isocitrate dehydrogenase.

GSH contributes significantly to maintenance of a reduced cellular environment. Under physiological conditions, a high reduced (GSH):oxidised glutathione (glutathione disulphide (GSSG)) ratio is maintained. GSH, synthesised in the cytosol, is transported into the mitochondria by dicarboxylate and 2-oxoglutarate carriers (Palmieri 1994). Within the mitochondria, GSH is regenerated from GSSG by the enzyme GSH reductase with NADPH as a cofactor. GSH acts as an electron donor for the GPXs, members of the selenoprotein family, which incorporate selenocysteine in their enzymatic catalytic site. These selenocysteine residues are highly redox-reactive and GPXs play an important role in the reduction of H_2_O_2_ to water. Selenium content of the adrenal is high and selenium is preferentially retained in this organ during selenium deficiency (Behne & Hofer-Bosse 1984). Indeed, selenium deficiency results in a significant depletion of GPX activity and a reduction in steroidogenesis in an adrenal cell line (Chanoine *et al*. 2001). Eight GPX isoforms have been identified in humans, GPX1 being the major isoform in most tissues, present primarily in the cytosol but also in small amounts in the mitochondria. Our unpublished data shows especially high expression in the adrenal cortex. In the testes and spermatozoa, the mitochondrial isoform of Gpx4 is the most prevalent form, with its depletion causing male infertility in mice (Imai *et al*. 2009).

**Figure 3:**
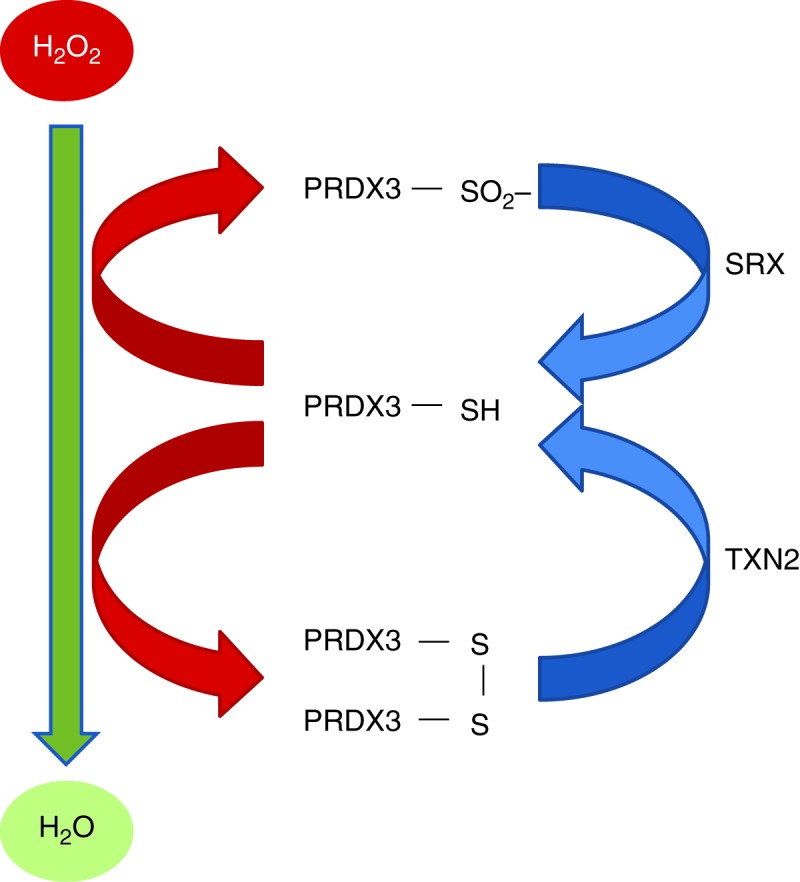
Maintenance of reduced peroxiredoxin 3 (PRDX3-SH) by thioredoxin 2 (TXN2) and sulfiredoxin (SRX). PRDX3, in its reduced form, detoxifies H_2_O_2_ in the mitochondria. This process induces the formation of disulphide PRDX3 which in turn is reduced back to PRDX3-SH by TXN2. With excessive H_2_O_2_ PRDX3 is hyperoxidised to an inactive sulfinic form (PRDX3-SO_2_^−^) and this hyperoxidisation is reversed by SRX.

Mammalian PRDXs also catalyse the reduction of H_2_O_2_ and lipid peroxides, albeit less efficiently than the GPXs. They comprise a family of six members, most of which (PRDX1–5) use TXN as an electron donor. PRDX3 and PRDX5 are present within the mitochondria; PRDX3 is mitochondria-specific and is seen in abundance particularly within the adrenal cortex, with a significant role in H_2_O_2_ detoxification within the mitochondrial matrix. It is estimated that PRDX3 is the catalyst for up to 90% of H_2_O_2_ generated within the matrix (Cox *et al*. 2010). During H_2_O_2_ elimination, two reduced PRDX3 subunits are converted to an oxidised disulphide-linked dimer that is reduced again by the mitochondrial TXN2 system (Zhang *et al*. 2007; Figs 1 and 3). TXN2 in turn is maintained in reduced form by the mitochondrial selenoprotein, thioredoxin reductase 2 (Fig. 1). In parallel, GSH can work together with glutaredoxin 2 in the mitochondria to also reduce PRDXs (Hanschmann *et al*. 2010) (Fig. 1).

The catalytic Cys in the mammalian 2-Cys PRDX enzymes (PRDX1–4) can undergo hyperoxidation to Cys sulfinic acid (Cys-SO_2_^−^), inactivating its peroxidase function (Woo *et al*. 2003). This inactive sulfinic form can be reduced back to the active form by sulfiredoxin, also seen most abundantly within the adrenal gland (Kil *et al*. 2012; Fig. 3). The only PRDX to be seen in sulfinic form within the adrenal cortex is PRDX3 and this inactivation of PRDX3 has recently been demonstrated to trigger a series of events within the adrenocortical environment (Kil *et al*. 2012). In both the murine and bovine models, adrenocorticotropic hormone (ACTH) stimulation increases inactivated sulfinic PRDX3, an effect that is prevented by treatment with metyrapone, a P450c11β (CYP11B1) inhibitor. This indicates that H_2_O_2_ produced in this final step of cortisol synthesis is mainly responsible for this hyperoxidation of PRDX3. Inactivation of PRDX3 results in accumulation of H_2_O_2_ which can diffuse into the cytosol, activating p38 MAPK signalling pathways with subsequent suppression of STAR protein synthesis and inhibition of steroidogenesis (Kil *et al*. 2012). It has been proposed that this mechanism of redox signalling exerts physiological control over steroidogenesis at the level of the adrenal gland, in addition to the well-characterised negative feedback exerted by cortisol on the hypothalamic–pituitary–adrenal (HPA) axis.

## Oxidative stress and steroidogenesis

Steroidogenesis significantly contributes to mitochondrial ROS production (Fig. 2) and in turn oxidative stress can impede steroidogenesis. STAR protein is essential for the transport of cholesterol across the mitochondrial membrane and is the first and rate-limiting step of steroidogenesis (Fig. 2). STAR is synthesised as a 37 kDa protein which is cleaved to a 30 kDa form in the mitochondria. The precise mechanism of cholesterol transfer across the mitochondrial membrane remains unclear. STAR is proposed to act in conjunction with a multi-component complex on the outer mitochondrial membrane. This complex includes the translocator protein (TSPO), voltage-dependent anion channel 1, TSPO-associated protein 7 (PAP7, ABCD3) and protein kinase A regulatory subunit 1α (PKAR1A) (Papadopoulos & Miller 2012). *STAR* mutations in humans have a clear effect on steroidogenesis with a phenotype ranging from severe lipoid congenital adrenal hyperplasia (LCAH), involving both adrenal and gonadal dysfunction to milder atypical or non-classic forms of LCAH which can resemble FGD (Baker *et al*. 2006, Metherell *et al*. 2009, Miller 2011). Mutations in other members of this outer mitochondrial membrane complex have, as yet, not been associated with human disease.

There is evidence that STAR is sensitive to both physiological and pathophysiological levels of ROS. Studies in Leydig cells, using H_2_O_2_ and agents such as perfluorododecanoic acid, demonstrate a clear reduction in the protein expression of the intramitochondrial (30 kDa) form of STAR, in response to ROS (Diemer *et al*. 2003, Shi *et al*. 2010). There is no clear consensus as to whether ROS also have an effect at a transcriptional level as current data are inconsistent. Evidence of an effect on other key components of the steroidogenic pathway such as 3β-hydroxysteroid dehydrogenase and P450c11β are also documented (Zhao *et al*. 2012, Prasad *et al*. 2013). P450c11 activity in cultured bovine adrenal cells and foetal human adrenocortical cells decreases rapidly as a result of oxidative damage and this inactivation may confer protection given the propensity of the P450c11β system to produce superoxide anions (Hornsby 1980). Recently, an additional mechanism by which steroidogenesis may be compromised by oxidative stress has been proposed in the Leydig cell model. STAR-mediated trafficking of redox-active cholesterol hydroperoxides results in mitochondrial toxicity, rendering steroidogenic cells susceptible to further oxidative insult (Korytowski *et al*. 2013).

## Oxidative stress and adrenal insufficiency in humans

Oxidative stress is implicated in several conditions characterised by primary adrenal failure including triple A syndrome, X-linked adrenoleukodystrophy (ALD) and most recently FGD. Despite ubiquitous expression of the proteins involved, in all three conditions defects in antioxidant defence specifically affect the adrenal. Mechanisms involving mitochondrial and peroxisomal dysfunction in addition to defective nucleocytoplasmic transport of DNA repair proteins and antioxidants demonstrate how disturbances in redox homeostasis in several subcellular compartments can have an effect on steroidogenesis.

## FGD and NNT deficiency

FGD is characterised by ACTH resistance and glucocorticoid deficiency, which may prove fatal if appropriate treatment with corticosteroids is not instigated. The majority of cases are caused by defects in ACTH signalling within the adrenal, namely mutations in the ACTH receptor, melanocortin 2 receptor (*MC2R*), and its accessory protein MRAP (melanocortin receptor accessory protein) (Clark *et al*. 1993, Metherell *et al*. 2005, Meimaridou *et al*. 2013). Novel mechanisms have recently been described in FGD, involving replicative and oxidative stresses (Hughes *et al*. 2012, Meimaridou *et al*. 2012). These include mutations in minichromosome maintenance protein 4 (*MCM4*), a DNA replicase and *NNT*, encoding NNT, a proton pump located in the inner mitochondrial membrane (Hughes *et al*. 2012, Meimaridou *et al*. 2012). NNT uses energy from the mitochondrial proton gradient to regenerate NADPH from NADP^+^, using NADH produced in the tricarboxylic acid cycle. NNT couples the production of NADPH to the rate of mitochondrial metabolism and the production of ROS generated by the electron transport chain. High concentrations of NADPH are required by both the GSH and TXN systems for detoxification of mitochondrial ROS. NADPH is also utilised by the electron transfer proteins ferredoxin reductase and ferredoxin, critical for the reduction of the P450 enzymes in steroidogenesis (Hanukoglu 2006). Thus there are several mechanisms by which NNT deficiency may affect steroidogenesis. NNT is widely expressed with high levels apparent in the adrenal gland in both human and mouse tissues (Meimaridou *et al*. 2012).

A substrain of C57BL6/J mice carries a spontaneous *Nnt* mutation leading to a 5-exon deletion; partial-deletion of the first domain of NNT, which binds NADH, and four of the 14 transmembrane domains which comprise the proton channel (Toye *et al*. 2005). This substrain of C57BL6/J mice shows an increased sensitivity to O_2_ · ^−^/ H_2_O_2_, particularly in the absence of mitochondrial MnSOD (SOD2) and a deficiency in both antioxidants results in death within 1–2 days (Huang *et al*. 2006) due to dilated cardiomyopathy. Introduction of a normal copy of *Nnt* into the *Sod2* ^−/−^ mice with the C57BL6/J background confers improved cardiovascular function during foetal development (Kim *et al*. 2010). Adrenals from the *Nnt*-mutant substrain have been noted to have slightly disorganised zona fasciculata with higher levels of apoptosis than WTs (Meimaridou *et al*. 2012). No differences in P450scc (CYP11A1) and P450c11β (CYP11B1) distribution were seen between the two substrains; however, the mutant mice had lower basal and stimulated levels of corticosterone, though the deficiency was not as marked as that seen in the human disease (Meimaridou *et al*. 2012). Lentiviral short hairpin RNA (shRNA)-knockdown of *NNT* in the human adrenocortical H295R cell line leads to increased levels of mitochondrial ROS, a decrease in the GSH:GSSG ratio and increased apoptosis (Meimaridou *et al*. 2012).

Extra-adrenal features have also been demonstrated in this substrain of mice. *Nnt*-mutant mice have impaired glucose tolerance with loss of glucose-dependent insulin secretion and ATP production in isolated pancreatic islet cells, reminiscent of a type 2 diabetes mellitus model (Kulkarni *et al*. 2003, Toye *et al*. 2005). When fed on a high-fat diet, C57BL/6J mice develop obesity, hyperglycaemia and insulin resistance (Rossmeisl *et al*. 2003). Interestingly, it has been demonstrated that whilst this strain does have reduced insulin secretion and impaired glucose tolerance in comparison to other strains of mice with higher expression levels of NNT, the C57BL/6J mice expressing the truncated protein have similar insulin secretion and glucose tolerance to the C57BL/6N mice expressing the full-length WT protein, though at lower levels than other strains (Wong *et al*. 2010). This indicates that it is low levels of NNT rather than the protein truncation caused by the 5-exon deletion that has an effect. This also raises the possibility that another enzyme is able to functionally compensate for the loss of NNT in the C57BL/6J mouse, this could explain the relative preservation of steroidogenesis in this strain in comparison to the human phenotype. C57BL/6J mice have also been shown to have a higher sensitivity to neurotoxic agents (Hamre *et al*. 1999). There are currently no published reports of extra-adrenal clinical manifestations in patients with *NNT* mutations; however, careful clinical surveillance is required for these patients given the ubiquitous expression of NNT.

## Triple A syndrome

Triple A syndrome (Allgrove syndrome) is a rare autosomal recessive disease characterised by the triad of alacrima, achalasia of the oesophageal cardia and primary adrenal failure (Allgrove *et al*. 1978). Alacrima is the earliest and most consistent sign with achalasia, occurring in about 75% of patients, the usual reason for referral. Adrenal insufficiency develops gradually over the first decade of life and may present later than the first two symptoms, but in some cases may be the presenting symptom leading to diagnosis of disease. This has led to the recommendation that in cases of the presence of alacrima and at least one more symptom of triple A syndrome, adrenal function testing and molecular analysis should be performed (Milenkovic *et al*. 2010). Glucocorticoid and adrenal androgen production are affected and a proportion of patients also go on to develop a mineralocorticoid deficiency. Neurodegenerative disease, features of which can include peripheral neuropathy, autonomic impairment, pyramidal and bulbar dysfunction, cerebellar and neuro-ophthalmological signs, occurs in ∼60% of patients (Houlden *et al*. 2002, Dixit *et al*. 2011, Vallet *et al*. 2012). In more than 90% of cases, the defect is due to mutations in the *AAAS* gene, with over 60 different mutations described in the literature. The phenotype is variable even within members of the same kindred and no genotype–phenotype correlation has been identified (Houlden *et al*. 2002, Huebner *et al*. 2002, Milenkovic *et al*. 2010).

The *AAAS* gene product is the 60 kDa nuclear pore complex (NPC) protein ALADIN (alacrima–achalasia–adrenal insufficiency neurologic disorder). *AAAS* mRNA and the ALADIN protein are ubiquitously expressed with predominance in the adrenal and CNS structures in the human and the rat (Handschug *et al*. 2001, Storr *et al*. 2005, Cho *et al*. 2009). ALADIN is the only nucleoporin to be associated with hereditary adrenal disease and the first to be associated with hereditary neurodegenerative disease. CNS disorders have since been described in two other nucleoporinopathies, resulting from mutations in *Nup62* and *RanBP2*/*Nup358*, although the precise pathogenic mechanisms are unclear (Basel-Vanagaite *et al*. 2006, Neilson *et al*. 2009, Neilson 2010). ALADIN's role at the NPC is unknown. Most naturally occurring *AAAS* mutations result in mislocalisation of the abnormal ALADIN protein (mainly into the cytoplasm), implying that correct NPC targeting is vital for its function (Krumbholz *et al*. 2006).

All the clinical features are progressive, suggesting a degenerative process. Patient adrenal histology reveals atrophy of the zona fasciculata and reticularis (Allgrove *et al*. 1978). Oxidative stress may play a role in the pathogenesis of this complex disease. Dermal fibroblasts of triple A patients have higher basal intracellular ROS and are more sensitive to oxidative stress than WT fibroblasts (Hirano *et al*. 2006, Kiriyama *et al*. 2008, Kind *et al*. 2010). Additionally in the dermal fibroblast model, failure of nuclear import of DNA repair proteins, aprataxin and DNA ligase I, and the antioxidant ferritin heavy-chain protein (FTH1) has been described (Hirano *et al*. 2006, Storr *et al*. 2009). How these nuclear import defects lead to an increase in intracellular ROS remains unclear. Increased chromosomal fragility has also been reported (Reshmi-Skarja *et al*. 2003), a finding that has also recently been described in cases of FGD within the Irish traveller community, caused by mutations in the DNA replicase *MCM4* (Hughes *et al*. 2012). Lentiviral-shRNA knockdown of *AAAS* in H295R human adrenocortical cells and SH-SY5Y human neuroblastoma cells renders them hypersensitive to oxidative stress, with a decrease in the GSH/GSSG ratio (Prasad *et al*. 2013). Furthermore, *AAAS* knockdown in H295R cells affects the key components of the steroidogenic pathway, STAR and P450c11β expression, with an effect on cortisol production, an effect that is partially reversed with antioxidant *N*-acetylcysteine treatment (Prasad *et al*. 2013).

*Aaas* ^−/−^ mice fail to show a similar phenotype to triple A syndrome patients and are largely indistinguishable from WT mice (Huebner *et al*. 2006). No significant difference in baseline ACTH and corticosterone measurements was observed. Histology revealed no gross abnormality of the organs and the adrenals, ovary, testes, oesophagus, pituitary and peripheral nerves had features similar to those of the WT. Infertility seen in the female *Aaas* ^−/−^ mice was postulated to be related to problems with the maturation of oocytes. Ultrastructurally, there were no observable differences in the NPC (using kidney and liver tissue; Huebner *et al*. 2006). Additionally, no NPC structural differences were detected by immunocytochemistry of embryonic dermal fibroblasts. These findings indicate the functional redundancy of ALADIN, at least in mice. ALADIN's function at the NPC may therefore be species-specific, certainly species-specific function of nucleoporins has been demonstrated with other Nups (Gigliotti *et al*. 1998, Grimaldi *et al*. 2007).

## X-linked ALD

X-linked ALD is an inherited neurometabolic disorder, incorporating progressive demyelination in the CNS, axonopathy in the spinal cord and adrenal insufficiency (Kemp *et al*. 2012). The disease is caused by mutations in the *ABCD1* gene on Xq28, encoding the ALD protein (ALDP), a member of the peroxisomal ATP-binding cassette (ABC) transporters. The disease is clinically heterogeneous and the clinical phenotype can be variable even within families. The three most common phenotypes reported are isolated adrenal insufficiency; a late onset slowly progressive disease adrenomyeloneuropathy in which two-thirds of patients also have adrenal insufficiency; and a cerebral inflammatory demyelinating form which can be fatal in early childhood (Kemp *et al*. 2012). Adrenocortical insufficiency can be the presenting symptom of adrenomyeloneuropathy years or even decades before the onset of neurological symptoms (Wichers-Rothers *et al.* 2005).

ALDP imports VLCFA-CoA esters into the peroxisome for degradation by β-oxidation (van Roermund *et al*. 2008). Peroxisomes play an important role in lipid metabolism and are in close communication with mitochondria. Human *ABCD1* expression is abundant in the adrenal gland, testis, liver, kidney, lung, placenta and intestine and this is mirrored in mouse expression profiles (Troffer-Charlier *et al*. 1998, Hoftberger *et al*. 2007). ALDP protein expression is particularly high in cells characterised by steroid hormone production (the adrenal gland and testis; Hoftberger *et al*. 2007). Within the adrenal gland, the highest expression is seen in the zona fasciculata and zona reticularis, reflective of the areas affected by the disease. Mutations in *ABCD1* result in abnormal accumulation of VLCFAs in plasma and various tissues, predominantly in myelin sheath, adrenal cortex and testis (Forss-Petter *et al*. 1997). Histology from both the mouse model and human specimens indicates the involvement of oxidative stress in the pathogenesis of the disease, with the evidence of increased MnSOD and lipid peroxidation (Powers *et al*. 2005). Increased ROS production, depletion of GSH and decreased mitochondrial membrane potential in patient dermal fibroblasts have been described (Fourcade *et al*. 2008). The precise mechanism whereby the accumulation of VLFCAs leads to the generation of free radicals is unclear and several effects have been proposed (Galea *et al*. 2012). VLCFAs are substrates of β-oxidation within the peroxisomes, a process that aids acyl-CoA availability and subsequent synthesis of plasmalogens, phospholipids with reported antioxidant properties (Hayashi & Oohashi 1995, Khan *et al*. 2008, Brites *et al*. 2009). Dysfunction within peroxisomes can have effects on mitochondria, as both organelles are in communication and generation of ROS within peroxisomes can disturb the mitochondrial redox balance (Ivashchenko *et al*. 2011). VLCFAs may also have direct effects on mitochondrial function, and structural abnormalities including lipid accumulation are seen within the mitochondria in *Abcd1* ^−/−^ mice (Forss-Petter *et al*. 1997). Diminished levels of NADH and ATP are seen within the spinal cord of *Abcd1* ^−/−^ mice, indicating defects in energy biosynthesis (Galino *et al*. 2011). Incorporation of VLFCAs into plasma membranes may also have deleterious effects affecting membrane properties and stability. In addition to effects on redox balance within peroxisomes and mitochondria, it has been proposed that VLCFAs may be involved in signalling within the immune system exacerbating inflammation, which in turn can have further effects on ROS and reactive nitrogen species (RNS) generation (Galea *et al*. 2012).

Mouse models for X-linked ALD have been established, involving knockout of the *ABCD1* gene. Elevated levels of VLCFAs are seen from 6 months of age in the nervous system and adrenal gland; however, there is no overt phenotype until 16 and 20 months of age, when locomotor alterations are evident (Forss-Petter *et al*. 1997, Pujol *et al*. 2002, 2004). The model is thus able to recapitulate some of the neurodegenerative aspects of the human disease. With regards to adrenal function, similar to the triple A syndrome mouse model, no effect is seen on corticosterone production at either baseline or upon stress, although histological changes including VLFCA accumulation and increased MnSOD expression are seen in the adrenal cortex (Forss-Petter *et al*. 1997, Lu *et al*. 2007).

## Models of disease

In all three adrenal insufficiency conditions discussed, mouse models of the disease do not faithfully recapitulate the human phenotype. It is possible that additional mechanisms of antioxidant defence exist within the adrenocortical environment of the mouse, conferring relative protection. Given the potential limitations of mouse models for studies of the adrenal aspects of these diseases, consideration should be given to other available *in vivo* models for further investigation of precise pathogenic mechanisms.

Non-human primate models such as *Papio hamadryas* (baboons) and *Macaca mulatta* (rhesus monkeys) have been used to study age-related changes in endocrine function (Goncharova & Lapin 2004). These have an advantage over rodents in that they produce DHEA/DHEAS, with a steroidogenic profile therefore more reflective of that in man. Similar to man, DHEA/DHEAS production decreases significantly whilst glucocorticoid production varies little with age (Ferrari *et al*. 2001, Goncharova & Lapin 2004). However, older monkeys exhibit reduced HPA axis sensitivity to glucocorticoid regulation, with a delay in normalisation of the axis after stimulation. This indicates impairment of negative feedback mechanisms, a phenomenon also observed in man. These non-human primate models have been used to study the effect of corticosteroids on antioxidant defence, in the context of ageing (Goncharova *et al*. 2008*a*,*b*, 2013). Circadian rhythmicity of SOD in erythrocytes correlates well with the characterised rhythmicity of DHEAS and cortisol (Goncharova *et al*. 2008*b*). Additionally, a similar age-related flattening of these SOD circadian rhythms has been observed, indicating a possible regulatory effect of corticosteroids on antioxidant defence. Age-related changes in stress responsiveness of SOD (increasing with age) and GSH reductase (decreasing with age) have also been described in non-human primate models (Goncharova *et al*. 2008*a*). This in turn can lead to peroxide oxidation of lipids, haemolysis of erythrocytes and a diminished reliability of oxygen transport. These studies indicate that corticosteroids themselves play a role in regulating the activity of antioxidants such as SOD and GSH reductase within erythrocytes.

## Conclusion

ROS play an important physiological role in steroidogenesis; however, in excess they can have deleterious effects on adrenal function. Steroidogenesis itself increases the generation of ROS, rendering the adrenal gland susceptible to oxidative damage. Inherited defects in the components of several different subcellular compartments have been described in association with increased susceptibility to oxidative stress and adrenal insufficiency. The use and further development of techniques pertaining to the detection of ROS in a site-specific manner will provide further information on the specific cellular targets involved. Further functional interrogation together with careful phenotyping of the respective patient cohorts will allow better understanding of the role of these proteins in human biology. Given the ubiquitous expression of the causative genes identified so far, this knowledge could be invaluable in directing patient surveillance and could also potentially provide targets for therapeutic intervention. Antioxidant defence within the adrenal certainly warrants further investigation and other components within this pathway may prove to be causative in the aetiology of as yet unaccounted for cases of primary adrenal insufficiency.
